# NF-*κ*B signaling in acute myocardial infarction: pathophysiology, inflammation, and therapeutic implications

**DOI:** 10.3389/fcvm.2026.1847945

**Published:** 2026-07-14

**Authors:** Yiyao Ma, Qingyun Jiang

**Affiliations:** Department of Emergency Medicine, Yixing People's Hospital, Yixing City, China

**Keywords:** acute myocardial infarction, cardiac remodeling, Inflammation, NF-*κ*B signaling, therapeutic strategies

## Abstract

Acute myocardial infarction (AMI) triggers a coordinated inflammatory and reparative response that determines infarct healing, ventricular remodeling, and long-term cardiac function. Nuclear factor-kappa B (NF-*κ*B) signaling is a central regulator of this process, but its role is highly context dependent. Early NF-*κ*B activation contributes to danger-signal recognition, immune-cell recruitment, and clearance of necrotic myocardium, whereas sustained activation promotes unresolved inflammation, cardiomyocyte death, fibroblast activation, and adverse ventricular remodeling. This dual function partly explains why experimental studies of NF-κB modulation in AMI have generated heterogeneous and sometimes conflicting results. In this review, we summarize the upstream triggers, temporal dynamics, cell-type-specific actions, and therapeutic implications of NF-*κ*B signaling in AMI. We also discuss the challenges of broad NF-*κ*B inhibition and highlight the need for temporally controlled and cell-selective strategies that preserve reparative inflammation while limiting chronic inflammatory injury and heart failure progression.

## Highlights

1. NF-*κ*B acts as a biphasic regulator in AMI, supporting early repair but driving adverse remodeling when persistently activated.
2. NF-*κ*B integrates DAMP sensing, inflammatory transcription, cardiomyocyte death, fibroblast activation, and ECM remodeling.
3. Precision NF-*κ*B modulation should prioritize time-specific, cell-selective, and network-based therapeutic strategies.

## 1 Introduction

Acute myocardial infarction (AMI) is a leading cause of morbidity and mortality worldwide, representing a major public health burden (1–5). The pathogenesis of AMI is driven primarily by the sudden rupture of an atherosclerotic plaque, which results in the formation of a blood clot that obstructs coronary blood flow (6–10). This ischemia, if prolonged, leads to myocardial injury, necrosis, and irreversible loss of heart muscle. Subsequently, the heart undergoes a complex series of pathological processes that include inflammation, cell death, and remodeling of the cardiac tissue (11). While these processes are crucial for repair, they can also contribute to the development of long-term complications, such as heart failure, arrhythmias, and further cardiac damage. The understanding of the molecular mechanisms underlying these events is critical to developing targeted therapeutic strategies for improving outcomes after AMI.

Central to the inflammatory response and tissue remodeling following AMI is the activation of the nuclear factor-kappa B (NF-*κ*B) signaling pathway. NF-*κ*B is a family of transcription factors, including p65, p50, RelB, and others, that regulate the expression of a wide range of genes involved in immune response, inflammation, cell survival, and apoptosis (12, 13). Under normal conditions, NF-*κ*B dimers are retained in the cytoplasm by inhibitors such as I*κ*B proteins. However, in response to various stimuli, such as cytokines (e.g., TNF-*α*, IL-1*β*), oxidative stress, and ischemia, I*κ*B proteins are degraded, allowing NF-*κ*B dimers to translocate into the nucleus and initiate transcription of target genes that promote inflammation and cellular stress responses (14–18). NF-*κ*B is thus a key mediator of the body's response to injury, including myocardial infarction. The role of NF-*κ*B in AMI is multifaceted and pivotal in both the acute inflammatory response and the subsequent healing process (19, 20). Activation of NF-*κ*B in cardiomyocytes and infiltrating immune cells triggers the release of pro-inflammatory cytokines, chemokines, and adhesion molecules that recruit additional immune cells to the infarcted area, exacerbating tissue damage (21, 22). NF-*κ*B also plays a critical role in cell death, particularly through apoptosis, necrosis, and pyroptosis, which contribute to the loss of viable heart muscle. Furthermore, NF-*κ*B signaling regulates cardiac remodeling by modulating extracellular matrix turnover, fibroblast activation, and fibrosis, which, if dysregulated, can lead to adverse ventricular remodeling and heart failure (23). Despite the beneficial aspects of NF-*κ*B activation in initiating repair mechanisms, its prolonged or excessive activation after AMI can lead to harmful outcomes. The inflammatory response, while essential for tissue healing, can also exacerbate myocardial injury and contribute to the development of pathological cardiac remodeling. Therefore, targeting the NF-*κ*B pathway presents an attractive therapeutic strategy for modulating the inflammatory and fibrotic processes that occur following AMI. Several pharmacological agents, including anti-inflammatory drugs and small molecule inhibitors, have shown promise in preclinical studies for their ability to inhibit NF-*κ*B activation and mitigate the deleterious effects of inflammation and remodeling.

Sterile inflammation is a non-infectious immune response triggered by endogenous danger signals released from injured or necrotic cardiomyocytes (24, 25). These danger-associated molecular patterns, including HMGB1, extracellular ATP, mitochondrial DNA, and heat shock proteins, activate innate immune pathways that are required for necrotic tissue clearance and infarct healing; however, if the inflammatory response fails to resolve in a timely manner, it may also promote excessive inflammation and adverse ventricular remodeling. AMI induces a form of sterile inflammation, in which immune activation is triggered by endogenous danger signals released from injured or necrotic cardiomyocytes rather than by infectious pathogens (26, 27). These danger-associated molecular patterns activate innate immune pathways and initiate inflammatory responses that are required for debris clearance and infarct healing, but their persistence may also contribute to excessive inflammation and adverse remodeling. Compared with recent high-level reviews in the field of post-AMI inflammation, including comprehensive discussions on biphasic inflammatory responses, multi-pathway signaling networks, and translational therapeutic strategies, this manuscript does not aim to provide another general overview of inflammatory signaling pathways in acute myocardial infarction. Instead, this review is specifically focused on NF-*κ*B as a temporal and cell-type–dependent regulatory switch that integrates ischemic stress signals and determines divergent cellular outcomes during AMI progression. Unlike previous reviews that primarily catalog signaling pathways or therapeutic agents, we emphasize the dynamic role of NF-*κ*B in coordinating the transition from early adaptive inflammation to sustained maladaptive remodeling. In particular, we highlight how NF-*κ*B functions differently across cardiomyocytes, macrophages, endothelial cells, and fibroblasts, thereby generating context-dependent outcomes that cannot be explained by pathway-level descriptions alone. This cell-specific and time-resolved framework provides a mechanistic explanation for the inconsistent results observed in NF-*κ*B-targeted therapeutic studies. This review emphasizes NF-*κ*B signaling as an integrative regulatory hub rather than a conventional inflammatory pathway component.

## 2 NF-*κ*B signaling pathway mechanisms

### 2.1 Comparison and interconnection with other inflammatory pathways

Post-AMI inflammation is regulated by multiple interconnected signaling pathways, including the NLRP3 inflammasome, TLR4/MyD88 signaling, JAK/STAT signaling, MAPK cascades, and TGF-*β*/SMAD-related fibrotic pathways (28–30). Although these pathways have been extensively reviewed, NF-*κ*B occupies a distinct position within this network because it functions not only as one downstream inflammatory pathway, but also as a transcriptional integration hub that connects upstream danger sensing with downstream cytokine production, inflammasome priming, immune-cell recruitment, cell-death regulation, and fibrotic remodeling (31, 32). For example, TLR4/MyD88 signaling is a major upstream route that activates NF-*κ*B in response to danger-associated molecular patterns released from necrotic cardiomyocytes (33). In contrast, the NLRP3 inflammasome is largely dependent on NF-*κ*B-mediated priming, because NF-*κ*B induces the transcription of NLRP3, pro-IL-1*β*, and other inflammasome-related components before inflammasome activation and pyroptotic amplification occur (34, 35). Similarly, JAK/STAT and MAPK pathways frequently cooperate with NF-*κ*B by reinforcing cytokine feedback loops and inflammatory transcriptional programs rather than replacing its central regulatory function (36). Therefore, the unique value of focusing on NF-*κ*B lies in its ability to explain how diverse inflammatory, oxidative, ischemic, and cytokine-derived signals are converted into coordinated transcriptional outputs during AMI progression. Unlike pathway-specific mediators that mainly regulate selected inflammatory or reparative processes, NF-*κ*B links multiple stages of post-infarction pathology, including early sterile inflammation, cardiomyocyte survival or death, macrophage activation, endothelial dysfunction, fibroblast stimulation, and adverse ventricular remodeling. This hub-like position also explains why broad NF-*κ*B inhibition may produce inconsistent results: blocking NF-*κ*B may suppress harmful chronic inflammation and fibrosis, but it may also interfere with early reparative inflammation and cell survival. Thus, NF-*κ*B should be understood as a temporal and cell-type-dependent regulatory switch within the post-AMI inflammatory network, rather than as a conventional linear inflammatory pathway. The distinct position of NF-*κ*B relative to other major inflammatory pathways in AMI is summarized in Table 1.

**Table 1:** NF-*κ*B pathway activation in acute myocardial infarction.

Pathway/Mechanism	Key Components/Signals	Role in AMI	Therapeutic Implications
TLR4/MyD88 Pathway	TLR4, MyD88, IRAK, IKK, NF-*κ*B	Activation by DAMPs leads to NF-*κ*B activation, promoting inflammation and immune cell recruitment to the infarcted area.	Targeting TLR4 or MyD88 to inhibit NF-*κ*B activation could reduce inflammation.
TNF Receptor Pathway	TNF-*α*, TNFR1, TRADD, IKK, NF-κB	Activation by TNF-*α* induces NF-κB activation, amplifying the inflammatory response and contributing to tissue damage.	TNF-*α* inhibitors (e.g., Etanercept) have potential to modulate inflammation.
IL-1 Receptor Pathway	IL-1*β*, IL-1R, MyD88, IKK, NF-κB	IL-1*β* binding to IL-1R triggers NF-κB activation, exacerbating inflammation and promoting cardiac injury.	IL-1*β* antagonists (e.g., Anakinra) could reduce inflammatory damage in AMI.
JAK/STAT Pathway	IL-6, JAK1/2, STAT3, NF-κB	IL-6-induced activation of JAK/STAT pathway cooperates with NF-κB to regulate cytokine production and enhance fibrosis.	Combining JAK inhibitors with NF-κB inhibitors could limit inflammation and fibrosis.
MAPK Pathway	p38 MAPK, JNK, ERK, IKK, NF-κB	MAPK activation enhances NF-κB signaling, promoting inflammation and apoptosis.	Targeting both MAPK and NF-κB pathways may improve therapeutic efficacy.

### 2.2 NF-*κ*B pathway activation

The activation of the NF-*κ*B signaling pathway is a critical event in various biological processes, including inflammation, immune response, cell survival, and tissue remodeling (37, 38). NF-*κ*B, a family of transcription factors, is typically kept in an inactive state in the cytoplasm by inhibitory proteins called I*κ*Bs. These I*κ*B proteins prevent the NF-*κ*B dimers from translocating into the nucleus by masking their nuclear localization signals. Upon cellular activation by various stimuli, including pro-inflammatory cytokines, pathogens, or cellular stress, I*κ*Bs are phosphorylated by the I*κ*B kinase (IKK) complex, leading to their degradation via the proteasome (39, 40). This degradation releases NF-*κ*B dimers, such as p65/p50, enabling them to translocate into the nucleus and initiate the transcription of target genes that mediate immune responses, inflammation, and cell survival.

One of the most well-characterized pathways that activate NF-*κ*B is the TLR4/MyD88 signaling pathway (33, 41, 42). The recognition of pathogen-associated molecular patterns (PAMPs), such as lipopolysaccharides (LPS) from bacteria, by Toll-like receptor 4 (TLR4) initiates this pathway. The binding of LPS to TLR4 recruits the adapter protein MyD88, which is essential for downstream signaling (43, 44). This complex then activates IL-1 receptor-associated kinase (IRAK), which leads to the phosphorylation and activation of the IKK complex. IKK then phosphorylates I*κ*B proteins, marking them for degradation, and thereby releasing the NF-*κ*B dimers (e.g., p65/p50) for nuclear translocation and transcriptional activation of target genes.

Besides the TLR4/MyD88 pathway, other significant pathways also contribute to the activation of NF-*κ*B. For instance, the TNF receptor (TNFR) pathway is another critical pathway that triggers NF-*κ*B activation (45). When TNF-*α* binds to its receptor TNFR1, it recruits adapter proteins, such as TRADD and RIPK1, which activate downstream signaling cascades involving IKK. This leads to the degradation of I*κ*B proteins and the activation of NF-*κ*B, promoting inflammatory gene expression. Similarly, the IL-1 receptor (IL-1R) signaling pathway also activates NF-*κ*B in response to cytokines like IL-1*β* (46–48). The binding of IL-1*β* to IL-1R recruits the adapter protein MyD88, which activates IRAK family members and, through the IKK complex, induces the phosphorylation of I*κ*B and the release of NF-*κ*B dimers. The NOD-like receptor (NLR) pathway, activated by intracellular pathogens or damage-associated molecular patterns (DAMPs), is another important NF-*κ*B activation route. NLRs, such as NOD1 and NOD2, recognize microbial components or endogenous danger signals and activate a cascade that involves the IKK complex. This pathway plays a key role in host defense and inflammatory responses. Lastly, the cytokine receptor signaling pathways, including the IL-6/JAK/STAT pathway, often intersect with NF-*κ*B signaling. For example, IL-6 activates the JAK/STAT pathway, which can synergistically promote NF-*κ*B activation and amplify inflammatory responses (49).

In AMI, NF-*κ*B activation is driven predominantly by ischemia-induced sterile injury rather than infection (50). Necrotic and stressed cardiomyocytes release distinct damage-associated molecular patterns (DAMPs), including HMGB1, extracellular ATP, mitochondrial DNA (mtDNA), and heat shock proteins (HSPs), which activate NF-*κ*B through partially overlapping but non-identical pattern-recognition receptor (PRR) pathways (51). HMGB1 can engage TLR2, TLR4, and RAGE, leading to MyD88-dependent activation of IRAK–TRAF6–TAK1 signaling and subsequent IKK-mediated degradation of I*κ*B*α* (52). Extracellular ATP mainly signals through purinergic receptors such as P2X7 and P2Y receptors, thereby promoting calcium influx, potassium efflux, inflammasome priming, and NF-*κ*B-dependent inflammatory transcription (53, 54). mtDNA released from injured mitochondria can be sensed by endosomal TLR9 through MyD88-dependent signaling or by cytosolic DNA-sensing pathways such as cGAS–STING, both of which may converge on NF-*κ*B activation (55, 56). HSPs, particularly HSP60 and HSP70, may act as endogenous danger signals through TLR2/TLR4-associated adaptor proteins, thereby reinforcing IKK–I*κ*B*α*–p65/p50 signaling in cardiomyocytes, endothelial cells, and infiltrating immune cells (57, 58).

**Figure 1:**
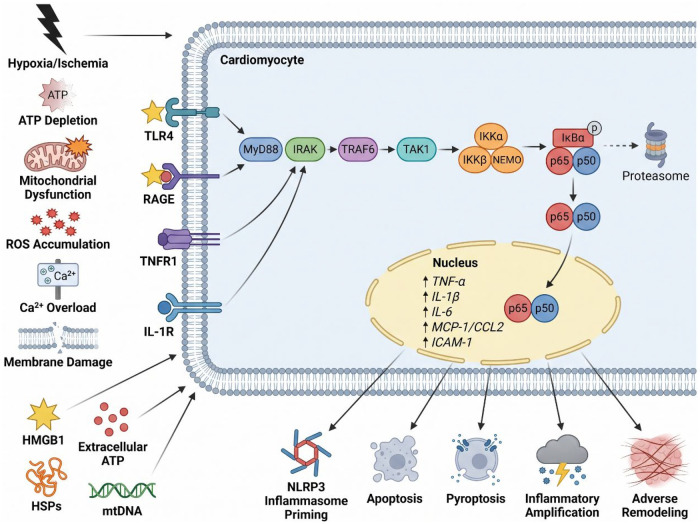
Hypoxic/ischemic activation of NF-*κ*B signaling in cardiomyocytes during acute myocardial infarction. Under hypoxic/ischemic stress, cardiomyocytes undergo ATP depletion, mitochondrial dysfunction, ROS accumulation, Ca^2^⁺ overload, and membrane injury, leading to the release of danger-associated molecular patterns, including HMGB1, extracellular ATP, HSPs, and mtDNA. These signals activate TLR4, RAGE, TNFR1, and IL-1R-related pathways, which converge on the MyD88–IRAK–TRAF6–TAK1–IKK axis. IKK-mediated phosphorylation and degradation of I*κ*B*α* releases NF-*κ*B p65/p50 dimers, allowing their nuclear translocation and transcriptional activation of inflammatory cytokines, chemokines, adhesion molecules, inflammasome-priming genes, and cell-death-related programs. This cardiomyocyte-centered response links ischemic injury to inflammatory amplification and adverse post-infarction remodeling.

Importantly, these DAMP–PRR axes do not simply produce a uniform NF-*κ*B response. Instead, the nature of the upstream DAMP, the receptor engaged, the adaptor proteins recruited, and the responding cell type collectively shape downstream transcriptional outputs (59). HMGB1–TLR/RAGE signaling preferentially amplifies cytokines, chemokines, and adhesion molecules that promote leukocyte recruitment and endothelial activation (60, 61). ATP–P2X7-related signaling more strongly couples NF-*κ*B priming to NLRP3 inflammasome activation, IL-1*β*/IL-18 maturation, and pyroptotic amplification (62, 63). mtDNA-triggered TLR9 or cGAS–STING signaling may generate a broader inflammatory and interferon-associated transcriptional program (55). HSP-mediated TLR activation may integrate stress-response and inflammatory signals, thereby influencing both cell survival and immune activation. Thus, NF-*κ*B activation in AMI should be understood as a DAMP-specific and receptor-context-dependent transcriptional program rather than a generic inflammatory response. This framework helps explain how ischemic sterile injury produces heterogeneous downstream outcomes, including cytokine release, immune-cell recruitment, inflammasome priming, cardiomyocyte death, endothelial dysfunction, and subsequent fibrotic remodeling (Figure 1). The major DAMP–PRR axes that activate NF-*κ*B during ischemia-induced sterile inflammation are summarized in Table 2.

**Table 2:** Therapeutic strategies targeting NF-κB in AMI.

Therapeutic Approach	Mechanism of Action	Current Evidence	Potential Benefits in AMI
Small Molecule Inhibitors	Inhibit IKKβ or p65, blocking NF-κB activation	BAY 11-7082, parthenolide (preclinical studies)	Reduces myocardial infarct size, decreases inflammation, and limits fibrosis.
Monoclonal Antibodies (e.g., Anti-TNF-α)	Block TNF-α and prevent NF-κB activation through TNFR blockade	Etanercept, Infliximab (clinical trials in inflammatory diseases)	Reduces systemic inflammation, improves cardiac function, and may prevent heart failure.
Natural Compounds (e.g., Tanshinone IIA)	Inhibit IKKβ and block NF-κB signaling	Tanshinone IIA, quercetin (preclinical studies)	Reduce infarct size, limit fibrosis, and promote myocardial repair.
Gene Therapies (e.g., siRNA, miRNA)	Target NF-κB-related genes (IKKβ, p65, TRAF6) to reduce activation	siRNA targeting IKKβ and p65 (preclinical studies)	Provide precise inhibition of NF-κB, with minimal off-target effects.
Mesenchymal Stem Cell (MSC) Therapy	Modulate inflammation via paracrine signaling, affecting NF-κB	Preclinical studies show MSCs can reduce NF-κB activation in AMI models	Promotes myocardial repair, reduces inflammation, and improves cardiac function.

### 2.3 NF-*κ*B downstream effects

NF-*κ*B plays a central role in regulating a wide variety of biological functions, including inflammation, immune response, cell survival, and cell death (64–66). One of the most prominent effects of NF-*κ*B activation is the induction of pro-inflammatory cytokines. Upon activation, NF-*κ*B dimers translocate into the nucleus and initiate the transcription of several key inflammatory mediators such as TNF-*α*, IL-6, and IL-1*β*. These cytokines are essential for immune cell recruitment to the site of injury or infection and for activating the adaptive immune response. The production of chemokines and cytokines such as MCP-1 (monocyte chemoattractant protein-1) further amplifies the inflammatory response (67–69). However, while NF-κB-mediated inflammation is critical for resolving infection and initiating tissue repair, sustained activation of NF-κB can lead to chronic inflammation, which contributes to tissue damage and the pathogenesis of inflammatory diseases like AMI.

In addition to its role in promoting inflammation, NF-κB is crucial for regulating cell survival through the upregulation of anti-apoptotic proteins. By increasing the expression of proteins like Bcl-2, Bcl-xL, and Mcl-1, NF-*κ*B helps prevent apoptosis, thereby enhancing cell survival in response to stress (70, 71). This mechanism is particularly important in the context of inflammatory diseases and cancer, where dysregulated NF-*κ*B activity promotes the survival of damaged or tumor cells. For example, in AMI, NF-*κ*B-mediated cell survival mechanisms may help preserve cardiomyocytes from apoptosis, contributing to tissue repair after myocardial injury.

**Figure 2:**
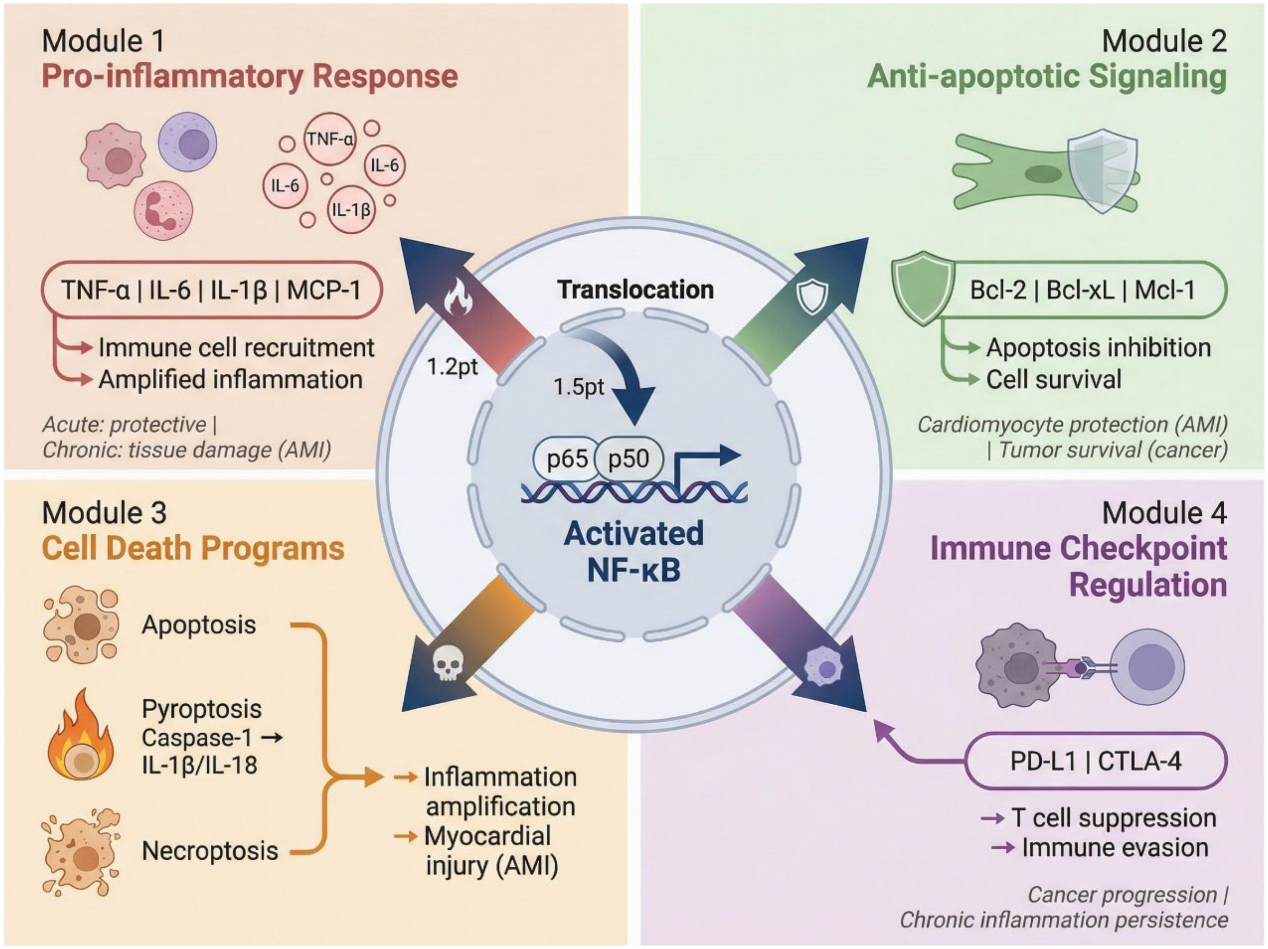
NF-*κ*B activation leads to the transcription of pro-inflammatory cytokines (TNF-*α*, IL-6, IL-1*β*), promoting immune cell recruitment. It also upregulates anti-apoptotic proteins (Bcl-2, Bcl-xL, Mcl-1) for cell survival. NF-*κ*B regulates cell death through apoptosis, pyroptosis (IL-1*β*, IL-18 release), and necroptosis. In cancer, NF-*κ*B induces immune checkpoint molecules (PD-L1, CTLA-4) to evade immune detection, contributing to chronic inflammation and tumor progression.

NF-*κ*B also regulates multiple forms of cell death, including apoptosis, pyroptosis, and necroptosis, all of which are critical in the context of AMI and other diseases. While NF-*κ*B promotes cell survival through anti-apoptotic proteins, it can also drive cell death in certain situations. NF-*κ*B can induce apoptosis by activating pro-apoptotic genes, such as FasL and TRAIL, in immune cells (72, 73). Moreover, NF-*κ*B plays a significant role in pyroptosis, a form of inflammatory cell death associated with the activation of caspase-1 and the release of inflammatory cytokines like IL-1*β* and IL-18 (74). This process is particularly important in AMI, where excessive pyroptosis can exacerbate myocardial injury by further amplifying the inflammatory response. NF-*κ*B's role in necroptosis, a regulated form of necrosis, also contributes to inflammation and tissue damage, as this form of cell death leads to the release of cellular contents and the propagation of inflammation (75, 76). These molecules suppress the activity of cytotoxic T-cells, allowing tumor cells to evade immune detection and destruction. This immune evasion mechanism is also observed in chronic inflammatory diseases, where NF-*κ*B-mediated immune suppression can hinder the resolution of inflammation and promote disease progression. In the context of AMI, while NF-*κ*B activation is required for the initial immune response, excessive NF-*κ*B activity can lead to the persistence of an inflammatory environment that hinders tissue repair and contributes to adverse outcomes such as heart failure. Following activation, NF-*κ*B drives a broad transcriptional program that links inflammatory signaling with cardiomyocyte survival and regulated cell death. As summarized in Figure 2, NF-*κ*B induces pro-inflammatory cytokines, chemokines, anti-apoptotic molecules, and inflammasome-related mediators, thereby coordinating immune-cell recruitment, cell survival, apoptosis, pyroptosis, and necroptosis in the post-infarction myocardium.

## 3 The role of NF-*κ*B signaling in acute myocardial infarction (Ami)

### 3.1 Cardiac injury and inflammatory response

NF-*κ*B plays a central role in initiating and regulating the inflammatory response following acute myocardial infarction (AMI). When ischemia occurs, myocardial cells are deprived of oxygen, leading to cellular stress and the release of danger-associated molecular patterns (DAMPs), such as high mobility group box 1 (HMGB1) and heat shock proteins (77–80). These DAMPs activate pattern recognition receptors (PRRs) on both cardiomyocytes and infiltrating immune cells. Among the most important PRRs are Toll-like receptors (TLRs), particularly TLR4, which is activated by lipopolysaccharides (LPS) from damaged cell membranes or bacteria, as well as endogenous molecules released during tissue injury (81–84). The activation of TLR4 and other PRRs triggers downstream signaling that activates NF-*κ*B, which is kept inactive in the cytoplasm under normal conditions by its inhibitor proteins, I*κ*Bs.

Upon activation by PRR signaling, I*κ*B proteins are phosphorylated and degraded by the I*κ*B kinase (IKK) complex, leading to the release of NF-*κ*B dimers, such as p65/p50. These dimers then translocate into the nucleus, where they bind to specific DNA sequences in the promoter regions of pro-inflammatory genes. NF-*κ*B drives the transcription of several key inflammatory mediators, including TNF-*α*, IL-1*β*, IL-6, chemokines (e.g., MCP-1), and adhesion molecules (e.g., ICAM-1), which recruit immune cells to the site of injury. This acute inflammatory response is essential for combating infection, clearing damaged cells, and initiating repair processes.

Immune cells such as neutrophils, macrophages, and lymphocytes are among the first responders to the site of injury. Macrophages, in particular, play a crucial role in both exacerbating inflammation and resolving it. Upon activation, macrophages release a variety of pro-inflammatory cytokines, including TNF-*α*, IL-1*β*, and IL-6, that further amplify the inflammatory response, recruit additional immune cells, and increase vascular permeability. These cytokines can activate the NF-*κ*B pathway in neighboring cells, creating a positive feedback loop of inflammation. Furthermore, macrophages help clear necrotic cells and cellular debris, and they secrete growth factors that promote tissue repair and fibrosis. However, while NF-*κ*B-driven inflammation is essential for initiating tissue repair, its persistent or excessive activation can lead to maladaptive inflammation, exacerbating myocardial injury and hindering recovery. Prolonged activation of NF-*κ*B can result in chronic inflammation, which promotes the activation of additional immune cells, the release of matrix-degrading enzymes, and excessive production of fibrotic tissue (85–89). This sustained inflammatory response can lead to cardiac remodeling, characterized by collagen deposition, fibrosis, and structural changes in the heart that impair its function. Furthermore, excessive NF-*κ*B activation can lead to myocardial cell death, including apoptosis and necrosis, contributing to further loss of viable cardiac tissue.

**Figure 3:**
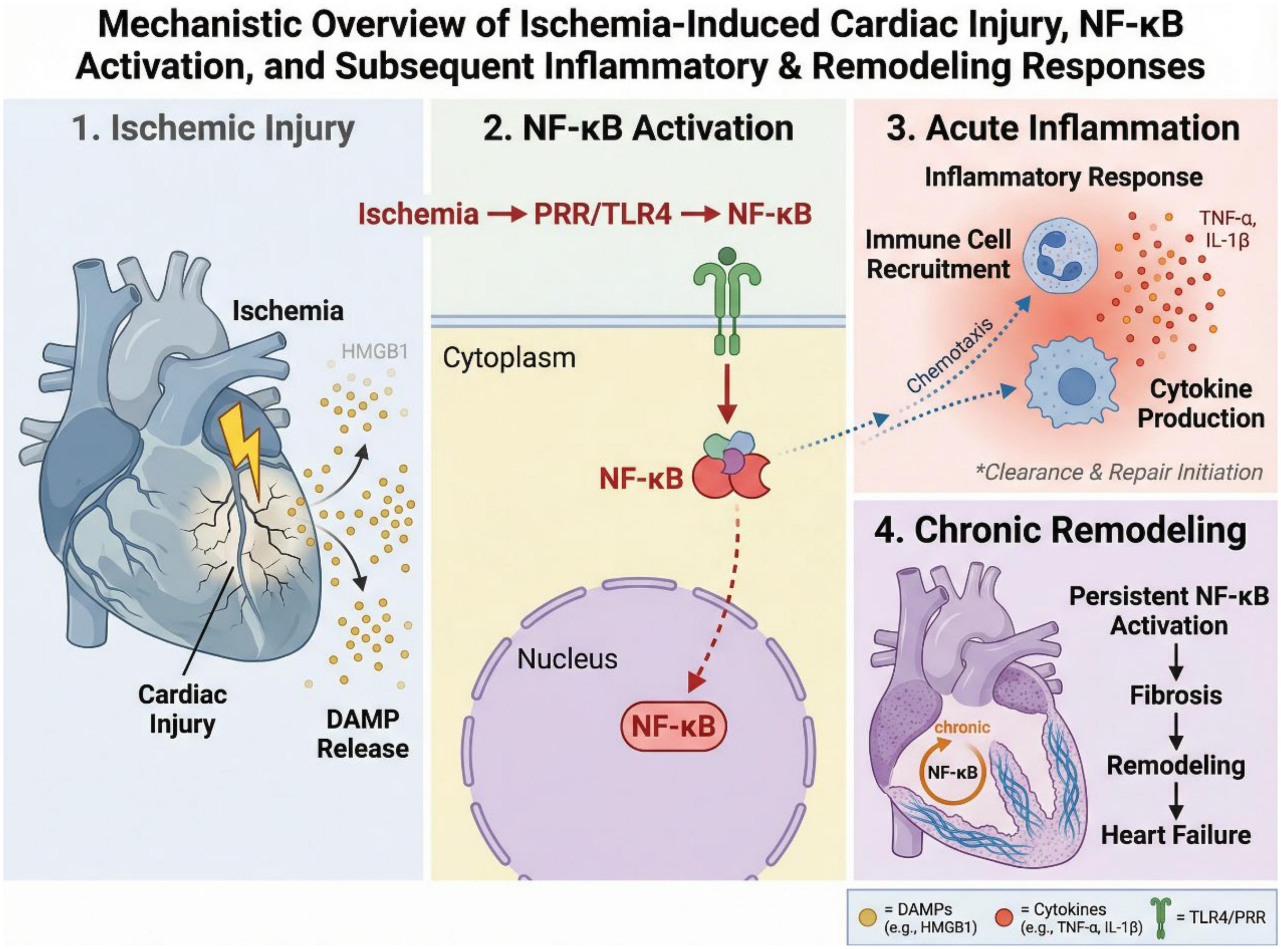
NF-*κ*B links ischemic injury to the inflammatory cascade in acute myocardial infarction (AMI). Ischemia-induced danger signals activate pattern recognition receptors, leading to NF-*κ*B nuclear translocation and transcription of pro-inflammatory mediators. Acute activation promotes immune cell recruitment, clearance of necrotic tissue, and initiation of repair. However, persistent NF-*κ*B activation sustains inflammation, drives fibrosis and adverse cardiac remodeling, and contributes to heart failure progression.

Studies have shown that inhibiting NF-*κ*B signaling in animal models of AMI can significantly reduce infarct size, limit inflammatory cell recruitment, and prevent the maladaptive effects of chronic inflammation (90–92). For example, NF-*κ*B inhibitors like BAY 11-7082 and parthenolide have been demonstrated to reduce the production of inflammatory cytokines, attenuate cardiac injury, and improve long-term cardiac function by limiting fibrosis and remodeling. This highlights the significant role that NF-*κ*B-driven inflammation plays in myocardial damage and remodeling after AMI. NF-*κ*B thus acts as a central hub that links ischemic injury to the inflammatory cascade, amplifying tissue damage while initiating the repair process (93). The balance between pro-inflammatory signaling and inflammation resolution is critical in determining the outcome of AMI. If inflammation is not properly resolved and NF-*κ*B remains persistently activated, it can lead to adverse cardiac remodeling and heart failure. On the other hand, timely and controlled activation of NF-*κ*B can promote effective tissue repair and recovery. Therefore, the regulation of NF-*κ*B activity is crucial in managing both the acute inflammatory response and the resolution of inflammation in AMI. In AMI, NF-*κ*B serves as a central molecular bridge between ischemic myocardial injury and the subsequent inflammatory cascade. As shown in Figure 3, transient NF-*κ*B activation supports immune-cell recruitment, necrotic tissue clearance, and early repair, whereas persistent activation sustains inflammation, promotes fibrosis, and contributes to adverse ventricular remodeling.

### 3.2 Initial vs. sustained Nf-*κ*B activation after Ami

NF-*κ*B signaling after AMI is highly time dependent, and the biological consequences of its activation differ substantially between the acute injury phase and the chronic remodeling phase. During the early phase of AMI, NF-*κ*B activation is mainly triggered by acute ischemic and hypoxic stress, mitochondrial dysfunction, oxidative stress, calcium overload, cardiomyocyte necrosis, and the release of danger-associated molecular patterns (DAMPs), including HMGB1, extracellular ATP, mitochondrial DNA, and heat shock proteins (94, 95). These signals activate pattern-recognition and cytokine receptors, such as TLR2/TLR4, RAGE, TNFR1, and IL-1R, which converge on the MyD88–IRAK–TRAF6–TAK1–IKK axis and promote I*κ*B*α* degradation and p65/p50 nuclear translocation (79, 96, 97). In this context, NF-*κ*B-induced expression of TNF-*α*, IL-1*β*, IL-6, CCL2/MCP-1, and adhesion molecules facilitates neutrophil and monocyte recruitment, promotes clearance of necrotic myocardium, and initiates the reparative response (95, 98, 99).

In contrast, sustained NF-*κ*B activation after the initial ischemic insult reflects a failure of inflammatory resolution rather than a simple continuation of the early danger response (94, 98). Several mechanisms may maintain chronic NF-*κ*B activity in the infarcted heart. First, incomplete clearance of necrotic debris can prolong DAMP-mediated TLR/RAGE activation (79, 96). Second, persistent mitochondrial dysfunction and ROS production can continue to stimulate IKK-dependent NF-*κ*B signaling (100). Third, cytokine feedback loops involving TNF-*α*, IL-1*β*, and IL-6 can reactivate NF-*κ*B through TNFR1, IL-1R, and JAK/STAT-related pathways (94). Fourth, NF-*κ*B-dependent priming of the NLRP3 inflammasome promotes IL-1*β* maturation, which in turn reinforces IL-1R–NF-*κ*B signaling (101). Fifth, impaired transition of macrophages from pro-inflammatory to reparative phenotypes can sustain cytokine and chemokine production (102, 103). Finally, post-infarction mechanical stress, neurohormonal activation such as angiotensin II signaling, and fibroblast-derived profibrotic mediators may further amplify NF-*κ*B activity during scar formation and ventricular remodeling (95, 104). Therefore, early NF-*κ*B activation mainly supports inflammatory recruitment and debris clearance, whereas sustained activation is maintained by self-amplifying inflammatory, oxidative, inflammasome-related, and fibrotic feedback loops that drive adverse remodeling and heart failure progression (94).

### 3.3 Myocardial cell death (apoptosis and pyroptosis)

Cell death is a hallmark of acute myocardial infarction (AMI) and significantly contributes to the loss of heart muscle, which impairs cardiac function (26, 105, 106). Two major forms of programmed cell death, apoptosis and pyroptosis, are particularly relevant in AMI. Both of these processes are regulated by the NF-*κ*B signaling pathway, which plays a central role in determining the extent of myocardial injury. While apoptosis typically helps eliminate damaged cells, pyroptosis is a more inflammatory form of cell death that exacerbates tissue damage and amplifies the inflammatory response.

Apoptosis, the process of programmed cell death, is essential for removing damaged cells, especially in the context of ischemia. NF-*κ*B regulates apoptosis by inducing the expression of pro-apoptotic genes such as Bax, caspase-3, and caspase-9, which are key players in the mitochondrial pathway of cell death. When activated, Bax promotes mitochondrial outer membrane permeabilization (MOMP), leading to the release of cytochrome c from the mitochondria (107, 108). This triggers the caspase cascade, ultimately resulting in cell fragmentation and death. The dual role of NF-*κ*B in apoptosis likely reflects differences in timing, stimulus intensity, cell type, and pathway crosstalk (71, 109). Transient NF-*κ*B activation during early ischemic stress may induce anti-apoptotic genes such as Bcl-2, Bcl-xL, c-IAPs, and survivin, thereby supporting cardiomyocyte survival (110, 111). In contrast, severe or sustained ischemia/reperfusion injury, persistent ROS production, calcium overload, mitochondrial dysfunction, and cytokine feedback can shift NF-*κ*B signaling toward a pro-death state by amplifying TNF-*α*/TNFR1, JNK/p38 MAPK, inflammasome priming, and caspase-related pathways (101, 112, 113). Thus, the pro- or anti-apoptotic outcome of NF-*κ*B activation depends on the duration, cellular context, and balance between survival and injury signals

While apoptosis is beneficial for clearing damaged cells, excessive activation of NF-*κ*B can lead to excessive myocardial cell loss, worsening cardiac dysfunction and contributing to adverse outcomes, including heart failure. In addition to apoptosis, pyroptosis is another form of programmed cell death that is particularly relevant in AMI. Unlike apoptosis, pyroptosis is associated with inflammation and the activation of caspase-1 (114–117). This enzyme cleaves and activates pro-inflammatory cytokines such as IL-1*β* and IL-18, which are subsequently released from the dying cells and contribute to an amplified inflammatory response. NF-*κ*B plays a pivotal role in regulating pyroptosis by upregulating inflammasome-related genes, such as NLRP3, which are involved in the activation of caspase-1 (34). The release of IL-1*β* and IL-18 further amplifies inflammation, recruits immune cells to the infarcted area, and accelerates tissue damage. In AMI, excessive pyroptosis contributes to a vicious cycle of myocardial injury, where inflammation and cell death perpetuate each other, hindering the healing process.

**Figure 4:**
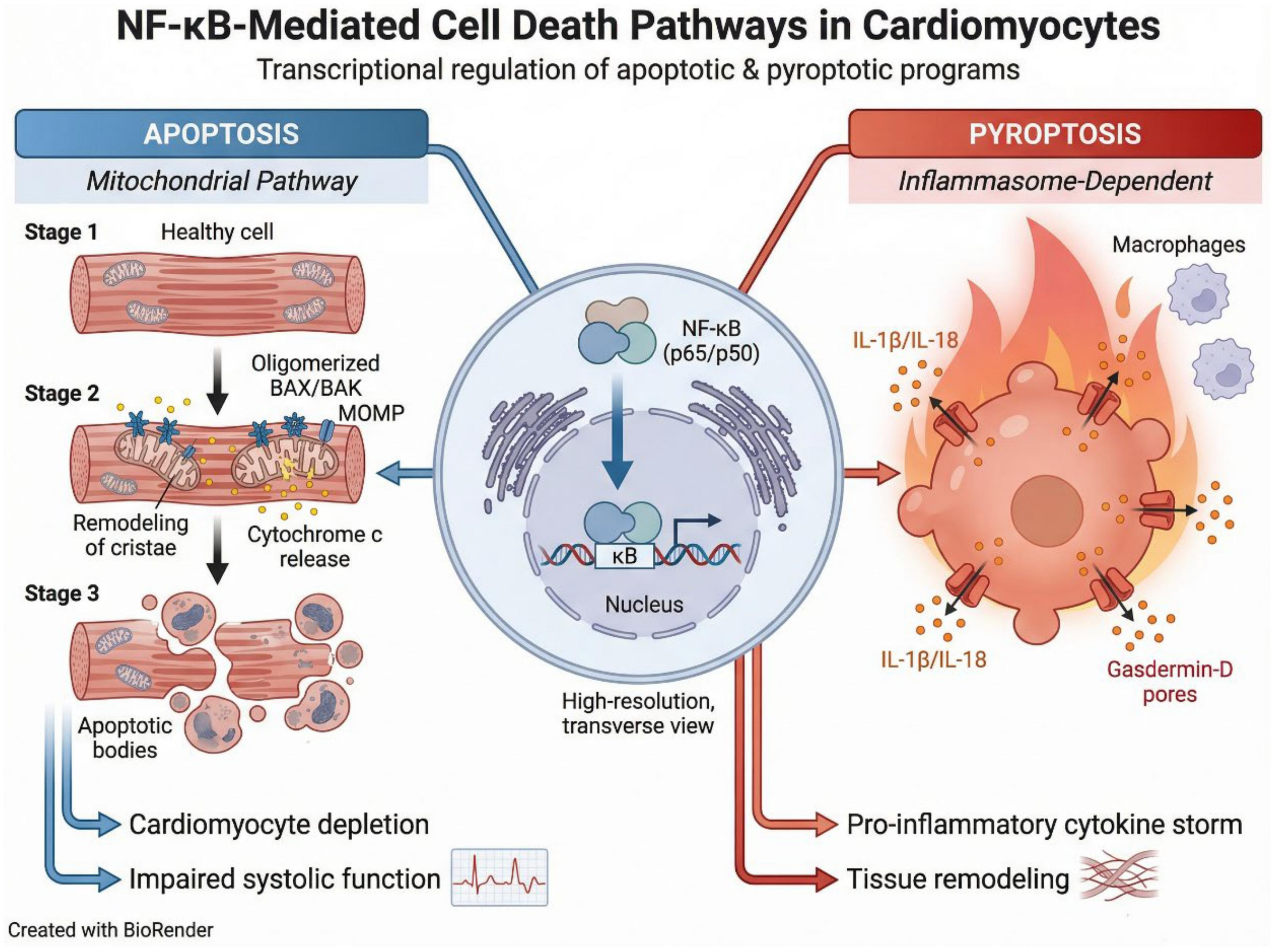
NF-*κ*B regulates myocardial cell death in acute myocardial infarction (AMI) by coordinating apoptosis and pyroptosis. Upon activation, NF-*κ*B drives transcriptional programs that promote mitochondrial apoptotic signaling and inflammatory cell death. Apoptosis contributes to cardiomyocyte loss and impaired contractile function, whereas pyroptosis amplifies inflammation through cytokine release. Excessive or persistent NF-*κ*B activation exacerbates myocardial injury, linking cell death to inflammatory amplification and adverse cardiac outcomes.

The relationship between NF-*κ*B activation and cell death is complex and context-dependent. While NF-*κ*B activation is necessary for the acute inflammatory response and tissue repair, excessive or prolonged NF-*κ*B signaling can lead to chronic inflammation and widespread cell death. Therefore, NF-*κ*B links ischemic stress to both apoptotic and inflammatory forms of cardiomyocyte death. Its contribution to apoptosis or pyroptosis may vary depending on the severity of ischemia, reperfusion status, mitochondrial injury, and the intensity of inflammasome priming. This context dependence may partly explain why NF-*κ*B inhibition has produced divergent effects across experimental AMI models. Beyond inflammatory cytokine induction, NF-*κ*B also contributes to myocardial injury by regulating programmed cell-death pathways in AMI. As illustrated in Figure 4, NF-*κ*B activation can promote mitochondrial apoptotic signaling and inflammasome-associated pyroptosis, thereby linking cardiomyocyte loss with inflammatory amplification and impaired cardiac function.

### 3.4 Cardiac remodeling and fibrosis

After the initial ischemic injury, the infarcted heart undergoes a dynamic remodeling process that includes inflammatory cell infiltration, fibroblast activation, extracellular matrix (ECM) deposition, scar formation, ventricular dilation, and functional decline (95). NF-*κ*B signaling contributes to this process not only by sustaining inflammatory cytokine production, but also by directly linking post-infarction inflammation to fibrotic remodeling (118). In the early reparative phase, controlled inflammation and scar formation are necessary to maintain ventricular integrity (119). However, persistent NF-*κ*B activation after the acute inflammatory phase may shift this process from physiological scar stabilization toward maladaptive fibrosis and adverse ventricular remodeling.

One important mechanism involves the regulation of TGF-*β*1-related profibrotic signaling. NF-*κ*B activation in macrophages, endothelial cells, fibroblasts, and stressed cardiomyocytes can promote the expression of inflammatory mediators that stimulate TGF-*β*1 production (119, 120). In turn, TGF-*β*1 activates downstream SMAD-dependent and non-SMAD pathways, leading to fibroblast activation, collagen synthesis, and ECM accumulation. This interaction places NF-*κ*B upstream of a major profibrotic cascade: sustained NF-*κ*B signaling maintains a cytokine-rich microenvironment that favors TGF-*β*1 induction, while TGF-*β*1 further reinforces fibroblast activation and matrix deposition. Therefore, NF-*κ*B should not be viewed only as an inflammatory regulator, but also as an upstream driver of inflammation-to-fibrosis transition after AMI.

NF-*κ*B also contributes to ECM remodeling by modulating the balance between matrix metalloproteinases (MMPs) and tissue inhibitors of metalloproteinases (TIMPs) (121–123). During early infarct healing, MMP activation facilitates degradation of damaged ECM and allows inflammatory cells and reparative cells to infiltrate the injured tissue. However, excessive or sustained NF-*κ*B activity may enhance the expression of MMPs such as MMP-2 and MMP-9, thereby promoting uncontrolled ECM degradation, ventricular wall thinning, and dilation (124, 125). Conversely, dysregulated TIMP expression can impair matrix turnover and favor excessive collagen accumulation. Thus, NF-*κ*B-mediated disturbance of the MMP/TIMP balance may contribute to both early matrix destabilization and later fibrotic stiffening, depending on the timing and intensity of pathway activation.

Another key mechanism is the involvement of NF-*κ*B in cardiac fibroblast-to-myofibroblast transdifferentiation (126). Under persistent inflammatory stimulation, NF-*κ*B-dependent cytokines and chemokines activate resident cardiac fibroblasts and promote their acquisition of a myofibroblast phenotype characterized by increased *α*-smooth muscle actin, collagen I/III production, contractile activity, and ECM-secreting capacity (127–129). These activated myofibroblasts are essential for infarct scar formation, but their prolonged persistence contributes to excessive fibrosis, ventricular stiffness, and impaired cardiac function. Therefore, the biological effect of NF-*κ*B in fibrosis is highly time dependent: transient activation supports necessary repair, whereas sustained activation maintains profibrotic fibroblast activity and drives maladaptive remodeling.

Taken together, NF-*κ*B promotes post-AMI remodeling through three interconnected mechanisms: induction of TGF-*β*1-related profibrotic signaling, disruption of MMP/TIMP-mediated ECM turnover, and promotion of fibroblast-to-myofibroblast transdifferentiation. This framework explains how unresolved inflammation is converted into structural remodeling and highlights why therapeutic strategies should aim to limit persistent NF-*κ*B-driven fibrotic programming without impairing early scar stabilization.

### 3.5 Long non-coding RNAs (lncRNAs) and Nf-*κ*B signaling interaction

**Figure 5:**
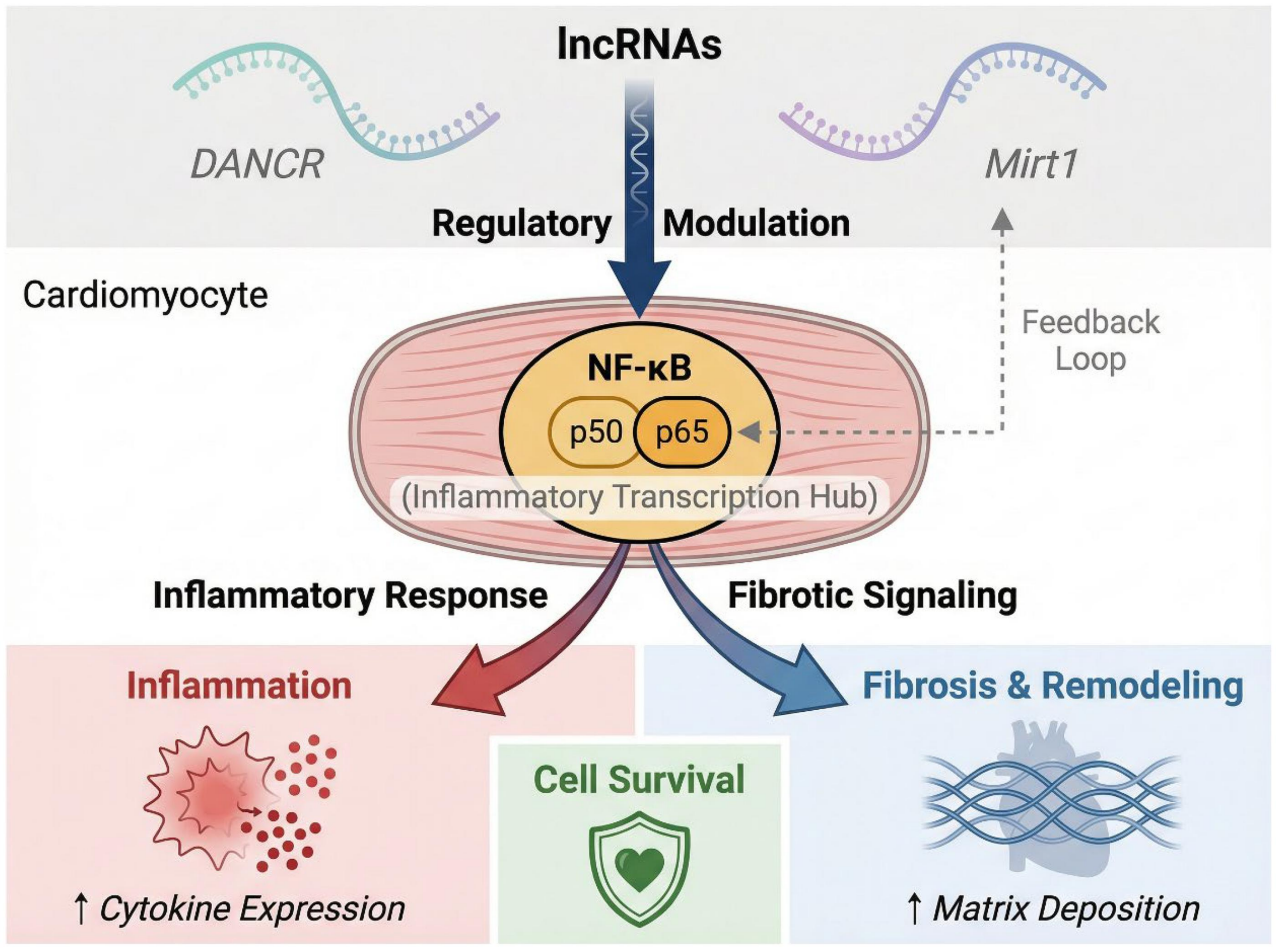
Long non-coding RNAs (lncRNAs) modulate NF-*κ*B signaling in acute myocardial infarction (AMI), fine-tuning inflammatory and fibrotic responses. By interacting with components of the NF-*κ*B pathway, lncRNAs such as DANCR and Mirt1 regulate transcriptional programs that control cytokine expression, cell survival, and extracellular matrix remodeling. Through this regulator*y* axis, lncRNAs influence the balance between adaptive repair and maladaptive inflammation, highlighting their potential as therapeutic targets and biomarkers in AMI.

Long non-coding RNAs (lncRNAs) represent an additional regulatory layer that can fine-tune NF-*κ*B signaling during AMI (130, 131). Rather than functioning as core inflammatory pathways themselves, lncRNAs mainly modulate the intensity, duration, and cellular specificity of NF-*κ*B-dependent transcription. Through interactions with transcription factors, chromatin-modifying complexes, microRNAs, or signaling proteins, selected lncRNAs may influence cytokine expression, cardiomyocyte survival, macrophage activation, fibroblast responses, and ECM remodeling (132–134). Several lncRNAs have been linked to NF-*κ*B-related regulation in ischemic myocardial injury. For example, DANCR has been reported to affect inflammatory gene expression and cardiomyocyte stress responses through NF-*κ*B-associated mechanisms (135). Mirt1 may also participate in ischemia-induced inflammation and cell injury by modulating NF-*κ*B activity and inflammatory cytokine production (136). These examples suggest that lncRNAs can shape NF-*κ*B output states rather than simply switching the pathway on or off. Therefore, lncRNAs should be viewed as context-dependent modulators of NF-*κ*B signaling in AMI. Their major value lies in refining the inflammatory and fibrotic response, potentially influencing the balance between adaptive repair and maladaptive remodeling (Figure 5). However, compared with the established roles of NF-*κ*B in cytokine induction, cell death, fibroblast activation, and ECM remodeling, lncRNA-related mechanisms remain relatively emerging. Further studies are needed to define their cell-specific functions, direct molecular targets, and translational feasibility in post-infarction remodeling.

## 4 Therapeutic targeting of NF*κ*B signaling

### 4.1 Evidence from small- and large-animal studies

Experimental studies evaluating NF-*κ*B signaling in AMI have produced heterogeneous and sometimes conflicting results across different animal models. In many small-animal studies, inhibition of NF-*κ*B-related inflammatory pathways, including TLR4/MyD88, TNF-*α*/NF-*κ*B, IKK-dependent signaling, and other upstream innate immune pathways, reduced inflammatory cytokine production, macrophage accumulation, cardiomyocyte apoptosis, fibrotic remodeling, and ventricular dysfunction (41, 47, 91, 92). These findings support the view that excessive or sustained NF-*κ*B activation contributes to maladaptive post-infarction inflammation and adverse ventricular remodeling (91, 92). However, other studies suggest that NF-*κ*B activation may also exert protective effects, particularly during the early ischemic phase. Cardiomyocyte NF-*κ*B signaling has been shown to protect against ischemia-induced apoptosis, indicating that early activation may support cell survival and tissue adaptation (71). This apparent contradiction highlights the time-dependent and cell-specific nature of NF-*κ*B signaling after AMI (47, 71, 91). Early NF-*κ*B activation may facilitate immune-cell recruitment, necrotic tissue clearance, and reparative signaling, whereas persistent activation may sustain cytokine production, inflammasome activation, fibroblast stimulation, and extracellular matrix remodeling (95, 102). Several factors may explain the inconsistent findings among animal studies. Permanent coronary ligation and ischemia/reperfusion models differ in the extent of necrosis, oxidative stress, calcium overload, and inflammatory amplification (137). Systemic pharmacological inhibition may affect cardiomyocytes, endothelial cells, immune cells, and fibroblasts simultaneously, whereas genetic or cell-specific approaches may reveal distinct roles of NF-*κ*B in individual cell types (71, 92). In addition, the timing of intervention is critical: early inhibition may interfere with reparative inflammation, whereas delayed inhibition may reduce chronic inflammation and fibrosis (47, 138). Large-animal studies remain limited, but pig models suggest that therapies promoting inflammatory resolution, rather than broadly suppressing NF-*κ*B, may have greater translational relevance (49). Representative small- and large-animal studies are summarized in Table 3.

**Table 3:** Representative small- and large-animal studies evaluating NF-κB-related mechanisms or interventions in acute myocardial infarction.

Study	PMID	Animal model	AMI/I/R model	NF-κB-related target or intervention	Main findings	Interpretation/relevance to conflicting evidence
Misra et al., 2003 (71)	14676146	Mouse	Acute myocardial infarction/ischemic injury	Cardiac NF-κB activation`	NF-κB protected adult cardiomyocytes against ischemia-induced apoptosis	Supports the concept that early or cardiomyocyte-intrinsic NF-κB activation may be protective, especially through anti-apoptotic signaling. This contrasts with studies showing benefit from NF-κB inhibition.
Gurantz et al., 2005 (139)	15949474	Rat	Post-myocardial infarction remodeling	Etanercept or intravenous immunoglobulin targeting inflammatory remodeling-related genes	Etanercept or intravenous immunoglobulin attenuated expression of genes involved in post-MI remodeling	Suggests that systemic modulation of TNF/NF-κB-related inflammatory signaling can alter remodeling, but the intervention was not NF-κB-specific.
Frantz et al., 2006 (121)	16837548	Mouse	Myocardial infarction	Genetic absence of NF-κB p50 subunit	Absence of p50 improved early survival and reduced left ventricular dilation after MI	Supports a detrimental role of excessive NF-κB signaling in post-MI heart failure progression; however, global p50 deficiency may affect multiple cell types.
Kawano et al., 2006	16632551	Mouse	Myocardial infarction	NF-κB blockade	NF-κB blockade reduced ventricular rupture, improved cardiac function, and improved survival after MI	Indicates that inhibiting excessive NF-κB activation can be beneficial in remodeling and heart failure; differs from Misra et al. because of different intervention strategy and biological context.
Onai et al., 2007 (91)	16920808	Rat	Myocardial infarction	IMD-0354-mediated inhibition of NF-κB activation	NF-κB inhibition improved LV remodeling and diastolic dysfunction; reduced fibrosis, macrophage accumulation, TGF-β1, MCP-1, MMP-9, and MMP-2	Supports the idea that post-acute NF-κB inhibition can reduce inflammatory and extracellular matrix remodeling. Infarct size was similar, suggesting effects were mainly on remodeling rather than initial injury.
Van Tassell et al., 2010 (47)	20125030	Mouse	Non-reperfused experimental AMI	Pharmacologic MyD88 inhibition	MyD88 inhibition prevented LV dilation and hypertrophy without significantly reducing infarct size	Shows that targeting upstream innate immune signaling can limit adverse remodeling while preserving infarct scar formation. This supports stage-selective modulation rather than complete NF-κB suppression.
Ma et al., 2012 (92)	22753411	Mouse	Myocardial infarction	Capn4 deficiency; reduced NF-κB signaling and inflammation	Capn4 deficiency inhibited NF-κB signaling, reduced inflammatory responses, remodeling, dysfunction, and mortality after MI	Suggests that calpain-dependent NF-κB activation contributes to adverse post-MI remodeling, but the model involves genetic modification and may not reflect acute pharmacological inhibition.
Zhang et al., 2013 (36)	23913709	Mouse	Global and regional myocardial ischemia/reperfusion injury	Cardiomyocyte-specific deletion of NF-κB p65	Cardiomyocyte-specific p65 deletion protected the injured heart, partly through preservation of calcium handling	Indicates that cell-specific NF-κB effects differ. Cardiomyocyte p65 may contribute to I/R injury, whereas other NF-κB components or immune-cell NF-κB may have distinct roles.
Sun et al., 2015 (41)	26668635	Rat	AMI model	BET inhibition through TLR4/TRAF6/NF-κB pathway	BET protein inhibition mitigated AMI damage and reduced TLR4/TRAF6/NF-κB activation	Supports anti-inflammatory NF-κB modulation in small-animal AMI models. However, BET inhibition is pleiotropic and not specific to NF-κB alone.
Gray et al., 2017	28077321	Mouse/*ex vivo* heart model	Myocardial ischemia/reperfusion	CaMKII*δ*-dependent regulation of NF-κB and TNF-α	CaMKII*δ* subtypes differentially regulated infarct formation through NF-κB and TNF-α	Highlights that upstream calcium-dependent signaling can modulate NF-κB differently depending on isoform and context, adding to mechanistic heterogeneity.
Han et al., 2018	29510381	Rat	Myocardial infarction	Qiliqiangxin; inhibition of TGF-β1/Smad3 and NF-κB signaling	Qiliqiangxin attenuated cardiac remodeling and reduced fibrosis-related signaling	Supports a role for NF-κB in fibrotic remodeling, but the compound has multi-target effects and cannot be interpreted as selective NF-κB inhibition.
Shi et al., 2021 (31)	34032295	Rat	AMI model	Astragaloside IV targeting TLR4/MyD88/NF-κB signaling	Astragaloside IV reduced inflammation and blocked TLR4/MyD88/NF-κB signaling in AMI	Supports the protective effect of inhibiting upstream TLR4/MyD88/NF-κB signaling. However, natural compounds often have antioxidant and metabolic effects beyond NF-κB.
Tesoro et al., 2022 (49)	36297479	Mouse and pig	Ischemia/reperfusion-related AMI model	NIL10, an IL-10 receptor-binding nanoparticle affecting STAT3/NF-κB-related inflammatory resolution	NIL10 improved cardiac function in mice and pigs, reduced inflammatory foci and fibrosis, and increased resolving M2-like macrophage populations	Important because it includes a large-animal pig model. It supports the idea that promoting inflammatory resolution, rather than broadly suppressing NF-κB, may be more translationally relevant.

### 4.2 Current NF-*κ*B inhibition strategies

The NF-*κ*B pathway has emerged as a critical therapeutic target for various inflammatory diseases, including acute myocardial infarction (AMI). Due to its central role in inflammation, cell death, and remodeling, inhibiting NF-*κ*B signaling holds significant therapeutic potential. Several strategies have been explored to block NF-*κ*B activation, including small molecule inhibitors, antibody therapies, and decoy strategies.

Small Molecule Inhibitors: The most widely studied small molecules targeting NF-*κ*B are those that inhibit the upstream components of the signaling pathway, particularly the IKK complex. BAY 11-7082 is a potent IKK*β* inhibitor that has shown promise in preclinical models by reducing NF-*κ*B activation, inflammatory cytokine production, and myocardial injury in AMI. Another example is parthenolide, a natural compound that inhibits IKK*β* and thereby blocks NF-*κ*B activation, offering potential benefits in controlling inflammation and fibrosis in AMI (51). In addition to IKK inhibitors, p65 inhibitors have also been developed to prevent the nuclear translocation and activation of NF-*κ*B dimers. These inhibitors target the DNA-binding domain of p65 or prevent the interaction between NF-*κ*B dimers and coactivators, thus blocking the transcription of inflammatory genes. Although these inhibitors have shown efficacy in preclinical studies, their clinical application remains limited due to issues with specificity and off-target effects.

Antibody Therapies: Antibodies targeting NF-*κ*B-related proteins have been investigated as potential therapies. Etanercept, an anti-TNF-*α* antibody, has been widely used to block TNF-*α*, a key cytokine involved in NF-*κ*B activation (139–141). By inhibiting TNF-*α* binding to its receptor, etanercept reduces downstream NF-*κ*B activation, thereby mitigating inflammation and improving outcomes in various inflammatory conditions, including AMI. Other monoclonal antibodies targeting cytokine receptors, such as anti-IL-1*β* antibodies, have shown potential in reducing myocardial inflammation and improving heart function after infarction. In clinical trials, NF-*κ*B inhibitors, particularly those targeting cytokine signaling, have demonstrated some benefits in reducing infarct size, improving cardiac function, and promoting tissue repair. However, the clinical translation of these inhibitors has been challenging due to the complexity of NF-*κ*B regulation and the need for precise modulation of this pathway without disrupting its essential roles in immune response and tissue healing.

### 4.3 The role of natural products and plant compounds

Natural products, particularly from plants, have long been explored for their potential in modulating NF-*κ*B signaling due to their bioactive components with anti-inflammatory properties. Tanshinone IIA, a compound derived from the roots of Salvia miltiorrhiza (Danshen), has been shown to effectively inhibit NF-*κ*B activation. Tanshinone IIA prevents the phosphorylation and degradation of I*κ*B proteins, thus blocking the nuclear translocation of NF-*κ*B dimers and reducing inflammation in myocardial injury (50). Studies, such as those by Chai et al. (2023), have demonstrated that tanshinone IIA treatment significantly reduces infarct size and improves cardiac function in animal models of AMI, highlighting its therapeutic potential.

Another class of compounds with potential anti-inflammatory and NF-*κ*B inhibitory effects are flavonoids, which are widely distributed in plants. Quercetin, a well-known flavonoid, has been shown to inhibit the NF-*κ*B signaling pathway by preventing I*κ*B degradation and inhibiting the activation of IKK (142). Quercetin and other flavonoids have demonstrated efficacy in reducing inflammation, myocardial damage, and fibrosis in AMI models. Feng et al. (2025) (23) highlighted the therapeutic promise of flavonoid compounds, suggesting that their antioxidant and anti-inflammatory properties could be harnessed to modulate NF-*κ*B signaling and reduce adverse outcomes in AMI. The potential of these plant-derived compounds lies in their dual role of inhibiting inflammation while promoting tissue repair, offering a promising therapeutic strategy for AMI. However, the clinical use of these natural compounds requires further investigation into their pharmacokinetics, bioavailability, and safety profiles, which are often challenges for plant-based therapeutics.

### 4.4 Gene therapy and cell-based therapies

Gene therapy and cell-based therapies represent an emerging frontier for targeting NF-*κ*B signaling in AMI. Gene editing techniques, such as CRISPR/Cas9, offer the potential to directly modify the expression of genes involved in NF-*κ*B activation. For example, knocking down or silencing genes encoding NF-*κ*B subunits (e.g., p65 or IKK*β*) in cardiomyocytes or immune cells can inhibit the inflammatory response and limit tissue damage after AMI. Furthermore, CRISPR/Cas9-mediated deletion of genes involved in the regulation of NF-*κ*B, such as TNF receptor-associated factors (TRAFs), could attenuate the downstream inflammatory effects of AMI and promote more efficient cardiac repair (143, 144). Cell-based therapies also hold promise in modulating NF-*κ*B signaling. Mesenchymal stem cells (MSCs) are widely studied for their regenerative potential, and recent evidence suggests that MSCs can regulate NF-*κ*B activity through paracrine signaling mechanisms (145–149). These cells secrete bioactive molecules that modulate inflammation and fibrosis, effectively “reprogramming” the local immune environment in the infarcted heart. Preclinical studies have demonstrated that MSC therapy can reduce NF-*κ*B-mediated inflammation, promote tissue repair, and improve cardiac function after AMI. Additionally, the use of small RNA molecules such as siRNA and miRNA to directly target NF-*κ*B-related genes represents a novel approach to regulating this pathway. miR-146a, a microRNA that inhibits NF-*κ*B signaling by targeting key molecules like TNF receptor-associated factor 6 (TRAF6), has shown promise in reducing inflammation and protecting against myocardial injury (150–153). Similarly, siRNA targeting IKK*β* or p65 can specifically inhibit NF-*κ*B activation, thus preventing inflammation, cell death, and fibrosis post-AMI. These RNA-based therapies offer a precise method of controlling NF-*κ*B activity and improving outcomes in AMI.

## 5 Future directions and challenges

### 5.1 Precise regulation of NF-*κ*B pathway

Achieving precise regulation of the NF-*κ*B signaling pathway in the treatment of acute myocardial infarction (AMI) presents both an opportunity and a significant challenge. NF-*κ*B plays a dual role in AMI: it is crucial for initiating the inflammatory response and tissue repair but can also contribute to excessive inflammation, fibrosis, and maladaptive remodeling when activated for prolonged periods. Therefore, strategies to modulate NF-*κ*B activity must aim to strike a delicate balance—enhancing its reparative functions while minimizing its pro-inflammatory effects.

One of the key challenges in targeting NF-*κ*B is selectivity. NF-*κ*B activation is involved in a wide array of biological processes, including immune responses, tissue healing, and cell survival. Broad inhibition of this pathway can lead to immunosuppression, increased susceptibility to infections, or impaired tissue repair. To address this, there is a growing interest in developing targeted inhibitors that can selectively block the overactivation of NF-*κ*B without disrupting its essential functions. For example, specific inhibitors targeting downstream components like p65 or IKK*β* are being explored, with the aim of inhibiting only the pathological activation of NF-*κ*B in inflamed tissues, such as the heart after AMI, while preserving its beneficial roles in other tissues. Another promising approach involves spatiotemporal control of NF-*κ*B inhibition. Using novel drug delivery systems, such as nanocarriers or targeted antibodies, researchers are exploring methods to deliver NF-*κ*B inhibitors directly to the infarcted tissue, thereby minimizing systemic side effects. Furthermore, RNA-based therapies, including siRNA and miRNA, offer the possibility of modulating NF-*κ*B at the genetic level, potentially allowing for a more precise and sustained therapeutic effect (154–158). Despite these advances, the development of specific NF-*κ*B inhibitors remains a major challenge. Given the complexity of NF-*κ*B signaling and its involvement in various physiological and pathological processes, designing inhibitors that can effectively target NF-*κ*B in the context of AMI without causing significant off-target effects will be a major hurdle. Additionally, issues such as bioavailability, stability, and tissue-specific delivery must be addressed before these therapies can be successfully translated into clinical practice.

### 5.2 Clinical translation and personalized therapy

The clinical translation of NF-*κ*B-targeted therapies is a promising yet challenging endeavor. While preclinical studies have demonstrated the efficacy of NF-*κ*B inhibitors in reducing myocardial injury, inflammation, and fibrosis, translating these findings to human patients requires a deep understanding of individual genetic and pathological variations. Personalized therapy, which takes into account a patient's genetic makeup, disease stage, and the specific molecular characteristics of their AMI, will be essential for optimizing NF-*κ*B-based treatments (52, 159).

Genetic background is one of the most important factors to consider in personalized therapy. For example, variations in NF-*κ*B pathway-related genes, such as TNF-*α* or IKK*β*, can influence an individual's response to inflammation and may impact the effectiveness of NF-*κ*B inhibitors. Similarly, polymorphisms in genes encoding for pro-inflammatory cytokines (such as IL-6 or IL-1*β*) may modify the patient's inflammatory response to AMI, requiring tailored therapies to modulate NF-*κ*B signaling more effectively (16, 160). Therefore, genetic screening could help identify patients who would benefit the most from NF-*κ*B inhibition and guide treatment decisions accordingly. Additionally, pathological type and disease progression will influence the therapeutic approach. Patients with different extents of myocardial damage (e.g., small infarcts vs. large infarcts) may require distinct interventions. For instance, those with more extensive tissue damage and advanced fibrosis may benefit from prolonged NF-*κ*B inhibition to reduce inflammation and prevent excessive fibrosis. Conversely, patients in the early stages of AMI may need more targeted therapies that promote tissue repair without disrupting the acute inflammatory response (161–163).

To enable the clinical application of NF-*κ*B inhibitors, biomarkers will be crucial for patient stratification. Identifying specific markers of NF-*κ*B activation or downstream inflammatory mediators in blood or tissue samples could help monitor the response to therapy and predict clinical outcomes. Moreover, combining NF-*κ*B inhibition with other targeted therapies, such as those that target fibrosis or cardiac remodeling, may enhance the overall therapeutic efficacy in AMI.

### 5.3 Interaction of NF-*κ*B with other signaling pathways

NF-*κ*B does not function as an isolated inflammatory pathway in AMI, but forms a highly interconnected signaling network with JAK/STAT and MAPK pathways. These pathways are activated by overlapping upstream stimuli, including DAMPs, cytokines, oxidative stress, ischemia/reperfusion injury, and neurohormonal activation. Their crosstalk is particularly important because it determines whether post-infarction inflammation remains transient and reparative or becomes sustained, self-amplifying, and maladaptive.

#### 5.3.1 Crosstalk between NF-*κ*B and JAK/STAT signaling

The JAK/STAT pathway is mainly activated by cytokines such as IL-6, IL-10, interferons, and other inflammatory mediators released after myocardial ischemic injury (36, 164). Among these mediators, IL-6-driven JAK2/STAT3 activation is particularly relevant to AMI because it participates in cardiomyocyte survival, immune-cell activation, fibroblast responses, and post-infarction remodeling (165, 166). NF-*κ*B and JAK/STAT signaling interact through several mechanisms. First, NF-*κ*B promotes the transcription of cytokines such as IL-6, TNF-*α*, and IL-1*β*, which can subsequently activate JAK/STAT signaling in cardiomyocytes, immune cells, endothelial cells, and fibroblasts. Second, activated STAT3 may cooperate with NF-*κ*B at the transcriptional level to amplify the expression of inflammatory cytokines, chemokines, and survival-related genes (167, 168). Third, cytokine feedback loops generated by NF-*κ*B and STAT3 can maintain inflammatory signaling after the initial ischemic insult, thereby contributing to persistent immune activation and ventricular remodeling. Thus, the NF-*κ*B–JAK/STAT axis should be viewed as a cytokine-driven amplification loop rather than two independent pathways. However, the biological consequence of this interaction is context dependent. During the early phase of AMI, JAK/STAT activation may exert cytoprotective effects by supporting cardiomyocyte survival and stress adaptation. In contrast, sustained NF-*κ*B–JAK/STAT signaling may prolong cytokine production, macrophage activation, endothelial dysfunction, and fibroblast stimulation. This duality indicates that combined targeting of NF-*κ*B and JAK/STAT should not aim at complete pathway blockade, but rather at limiting excessive cytokine-driven amplification while preserving early adaptive repair.

#### 5.3.2 Crosstalk between NF-*κ*B and MAPK signaling

MAPK pathways, including p38 MAPK, JNK, and ERK, are rapidly activated by ischemic stress, ROS accumulation, inflammatory cytokines, mechanical stretch, and reperfusion-related injury (169). These pathways interact with NF-*κ*B at multiple levels. Upstream kinases activated by stress and cytokine receptors can simultaneously stimulate MAPK cascades and the IKK–I*κ*B*α*–NF-*κ*B axis (87). In addition, p38 MAPK and JNK may enhance NF-*κ*B-dependent transcription by regulating transcription factor phosphorylation, coactivator recruitment, chromatin accessibility, and mRNA stability of inflammatory mediators (170). ERK signaling may also interact with NF-*κ*B in fibroblasts and cardiomyocytes, thereby influencing cell survival, proliferation, extracellular matrix production, and post-infarction fibrosis (171, 172). Functionally, the NF-*κ*B–MAPK interaction links sterile inflammatory signaling to stress-induced cell death and fibrotic remodeling. p38 MAPK and JNK activation may amplify NF-*κ*B-mediated expression of TNF-*α*, IL-1*β*, IL-6, CCL2, and adhesion molecules, thereby enhancing leukocyte recruitment and inflammatory injury (169). At the same time, MAPK signaling can promote apoptosis, fibroblast activation, and extracellular matrix remodeling, which may cooperate with sustained NF-*κ*B activity to drive adverse ventricular remodeling (172). Therefore, the NF-*κ*B–MAPK axis represents a major stress-responsive module connecting ischemia/reperfusion injury, inflammation, cell death, and fibrosis.

#### 5.3.3 NF-*κ*B, JAK/STAt, and MAPK as a convergent inflammatory network hub

Rather than operating as parallel linear cascades, NF-*κ*B, JAK/STAT, and MAPK signaling form a convergent inflammatory network hub in the infarcted myocardium. NF-*κ*B primarily provides the transcriptional backbone for cytokine, chemokine, adhesion molecule, and inflammasome-related gene expression (173, 174). JAK/STAT signaling translates cytokine stimulation into immune and survival-related transcriptional programs. MAPK pathways transmit oxidative, mechanical, cytokine-derived, and ischemia/reperfusion-related stress signals into inflammatory, apoptotic, and fibrotic responses (169, 170, 175). Their convergence creates a self-reinforcing circuit in which DAMPs and cytokines activate NF-*κ*B and MAPK, NF-*κ*B-induced cytokines activate JAK/STAT, and JAK/STAT or MAPK signaling further amplifies NF-*κ*B-dependent transcription (36, 168). This network-level view helps explain why single-pathway inhibition often produces incomplete or inconsistent effects in AMI models. Blocking NF-*κ*B alone may reduce inflammatory transcription but may not fully suppress cytokine-dependent JAK/STAT activation or stress-induced MAPK signaling (173, 175). Conversely, inhibition of JAK/STAT or MAPK may dampen selected downstream responses but may leave NF-*κ*B-mediated DAMP sensing and transcriptional priming intact. Therefore, the therapeutic value of targeting this network may depend on identifying the dominant inflammatory module in a given stage of AMI and applying temporally controlled, mechanism-based combination strategies.

#### 5.3.4 Combination targeting strategies: preclinical evidence and challenges

Preclinical studies suggest that targeting NF-*κ*B together with JAK/STAT or MAPK-related pathways may provide stronger anti-inflammatory and anti-remodeling effects than inhibition of a single pathway (36, 164). For example, modulation of the JAK2/STAT3/NF-*κ*B axis has been reported to reduce inflammatory responses and myocardial injury in AMI models (166, 176). Similarly, interventions that suppress ERK/MAPK or p38/JNK-related stress signaling may attenuate apoptosis, inflammation, fibrosis, and adverse remodeling, partly through reduced cooperation with NF-*κ*B-dependent transcription (171, 177). These findings support the rationale for combination strategies aimed at limiting cytokine amplification, oxidative stress responses, and fibrotic remodeling simultaneously. Nevertheless, several challenges must be addressed before such strategies can be translated clinically. First, NF-*κ*B, JAK/STAT, and MAPK pathways all have protective functions during the early repair phase, including cell survival, inflammatory clearance, angiogenesis, and scar stabilization. Excessive or premature inhibition may therefore impair infarct healing. Second, systemic combination inhibition may increase the risk of immunosuppression, infection, impaired tissue repair, or off-target toxicity. Third, the optimal timing, dosage, treatment window, and patient population remain unclear. Future strategies should therefore prioritize stage-specific and cell-selective modulation rather than broad suppression of the entire inflammatory network. Biomarker-guided stratification, targeted delivery systems, and short-course combination therapy may help maximize therapeutic benefit while minimizing disruption of reparative inflammation.

## 6 Conclusion

NF-*κ*B signaling is a central but context-dependent regulator of AMI pathophysiology. Its role cannot be defined simply as protective or detrimental. Early activation contributes to danger-signal sensing, immune-cell recruitment, and clearance of necrotic myocardium, whereas sustained activation promotes unresolved inflammation, cardiomyocyte death, fibroblast activation, and adverse ventricular remodeling. The major challenge for therapeutic translation is therefore not whether NF-*κ*B should be inhibited, but when, where, and to what extent it should be modulated. Future studies should prioritize time-resolved and cell-specific analyses, clarify discrepancies among animal models, and develop targeted strategies that preserve reparative inflammation while limiting chronic inflammatory injury and heart failure progression.
